# Assessing the Compliance of Physicians With the American Diabetes Association (ADA) Guidelines in Prescribing Cardioprotective Antihyperglycemic Agents to Diabetic Patients at a University Hospital in Jeddah, Saudi Arabia

**DOI:** 10.7759/cureus.44133

**Published:** 2023-08-25

**Authors:** Ahmed M Sheikh, Anas Sheikh, Amani Alhozali, Saleh A Alshaikhi

**Affiliations:** 1 Diabetes and Family Medicine, King Abdulaziz University Hospital, Jeddah, SAU; 2 Cardiology, King Abdulaziz University Hospital, Jeddah, SAU; 3 Endocrinology and Metabolism, King Abdulaziz University, Jeddah, SAU; 4 Family Medicine, Ministry of Health, Al Qunfudah, SAU

**Keywords:** heart failure, sodium-glucose co-transporter-2 inhibitors, glucagon-like peptide-1 receptor agonists, ada guidelines, atherosclerotic cardiovascular disease, cardiovascular disease, type 2 diabetes

## Abstract

Background and objective

Diabetes is a major risk factor for the development of cardiovascular diseases. To tackle this issue, guidelines have emphasized the use of cardioprotective antihyperglycemic agents [glucagon-like peptide-1 receptor agonists (GLP1-RAs) and sodium-glucose co-transporter-2 (SGLT2) inhibitors] among type 2 diabetic patients at high risk of/with established atherosclerotic cardiovascular disease (ASCVD) or heart failure to curb morbidity and mortality in such individuals. The aim of this study was to assess physicians' adherence to the American Diabetes Association’s (ADA) recommendations on the use of cardioprotective antihyperglycemic agents among such patients.

Methods

This study involved a retrospective records review of physicians' treatment plans for all type 2 diabetic patients aged 18 years and older and attending outpatient clinics from the year 2019 to 2022 at King Abdulaziz University Hospital, Jeddah, Saudi Arabia, who (a) were at high risk of/with established ASCVD as defined by the American Diabetes Association (ADA) or (b) had heart failure with reduced ejection fraction (HFrEF) and required cardioprotective antihyperglycemic agents.

Results

We reviewed physicians' treatment plans for 202 type 2 diabetic patients in this study. All patients were either at high risk of or had established ASCVD. In addition, 36 (17.8%) of these patients had HFrEF. Although all patients were candidates for cardioprotective antihyperglycemic agents, only 56.9% of them received treatment as recommended by the ADA guidelines.

Conclusion

Despite being suitable candidates for cardioprotective antihyperglycemics, a significant number of patients were not treated based on the ADA guidelines, and this demonstrates a lack of adherence to the guidelines by physicians.

## Introduction

Diabetes has been shown to be a major risk factor for the development of atherosclerotic cardiovascular disease (ASCVD) and eventual heart failure. Over the years, studies have reported that ASCVD - defined as coronary heart disease (CHD), cerebrovascular disease, or peripheral arterial disease of atherosclerotic origin - is a leading cause of morbidity and mortality among diabetic individuals [1]. Furthermore, the rates of hospitalizations due to incident heart failure have been reported to be twofold higher in diabetic as opposed to non-diabetic patients [2,3], with approximately 50% of those with type 2 diabetes at risk of developing heart failure [4]. Saudi Arabia ranks among the top 10 countries globally with regard to the prevalence of diabetes [5]. The prevalence of established cardiovascular disease and those at high risk of developing cardiovascular disease among type 2 diabetic patients in Saudi Arabia has been reported to be 18% and 13.2%, respectively [6].

In an effort to curb these alarming and fatal outcomes and the cardiovascular risk associated with some antihyperglycemic medications for type 2 diabetes, the FDA issued guidelines in 2008 on the need for cardiovascular outcome trials to assess the safety of new diabetes medications [7]. To date, numerous trials have been carried out to test cardiovascular outcomes and many have shown encouraging results. So far, most of these successful outcomes have been associated with classes of antihyperglycemics called glucagon-like peptide-1 receptor agonists (GLP1-RAs) and sodium-glucose co-transporter-2 (SGLT2) inhibitors [8]. Meta-analyses of these trials have shown the benefits of the use of SGLT2 inhibitors and GLP1-RA in type 2 diabetic patients at high risk of ASCVD or established ASCVD, as well as in heart failure patients with reduced ejection fraction with the former class of medication [9,10]. All SGLT2 inhibitors have been shown to reduce the risk of worsening heart failure and subsequent hospitalization and death, with empagliflozin, dapagliflozin, and canagliflozin additionally demonstrating a reduction in the risk of major adverse cardiovascular events [11-15]. On the other hand, GLP1-RAs - namely dulaglutide, liraglutide, and semaglutide - have been proven to reduce the risk of major adverse cardiovascular outcomes with a neutral effect on heart failure [16-19].

Based on these cardiovascular outcomes, the American Diabetes Association (ADA) Guidelines of 2018 and 2019 recommended the addition of SGLT2 inhibitors and GLP1-RAs, respectively, due to their demonstrated cardiovascular benefits, for the management of type 2 diabetic patients with cardiovascular diseases [20,21]. In 2021, the ADA further recommended their use independent of the patient’s baseline HbA1c level, individualized HbA1c target, or the initiation of metformin in patients with established ASCVD or indicators of high risk (including patients >55 years of age with coronary, carotid, or lower-extremity artery stenosis >50% or left ventricular hypertrophy) as well as the use of SGLT2 inhibitors in patients with heart failure, particularly those with a reduced left ventricular ejection fraction (LVEF) of <40% [heart failure with reduced ejection fraction (HFrEF)] [22]. Furthermore, not only do these medications impact the health of the patients by reducing morbidity and mortality, but their use also optimizes the utilization of costly resources for the care of such patients [1]. The magnitude of such a positive impact will inevitably be beneficial among populations with a high prevalence of diabetes, such as in Saudi Arabia [5].

Although several studies have been conducted in Saudi Arabia and elsewhere to evaluate the adherence of physicians to guidelines in general, a focused evaluation of the prescription of these cardioprotective agents has been seldom performed. It is thus imperative to evaluate the degree to which these guidelines are implemented for the benefit of the patients and the healthcare system in general. In light of this, this study aimed to assess the adherence of physicians to ADA recommendations in the prescription of SGLT2 inhibitors and GLP1-RAs - medications that offer proven cardiovascular benefits - to patients with type 2 diabetes and established ASCVD or those with indications of high ASCVD risk, as well as in the prescription of SGLT2 inhibitors to heart failure patients.

## Materials and methods

The design we adopted for this study involved a retrospective review of the records of physicians’ treatment plans for patients with type 2 diabetes who required cardioprotective antihyperglycemic agents and attended outpatient clinics from the year 2019 to 2022 at King Abdulaziz University Hospital, Jeddah, Saudi Arabia. A computer-generated list of the patients was created after obtaining the approval of the university’s Biomedical Ethics Research Committee. Patient confidentiality was maintained through the use of hospital computers dedicated to reviewing file records within the vicinity of the hospital dictation rooms.

All type 2 diabetic patients aged 18 years and older and registered at outpatient clinics with either established ASCVD or belonging to a high ASCVD risk category - defined by the ADA as patients >55 years of age with coronary, carotid, or lower-extremity artery stenosis >50% or left ventricular hypertrophy - and heart failure patients [specifically with preserved ejection fraction (pEF) >50% or reduced ejection fraction (rEF) <40%] were included in the study. Patients not meeting these criteria were excluded. The authors developed and employed a data collection sheet that consisted of three parts: demographic data, biochemical parameters, and an assessment of physicians’ adherence to ADA recommendations in treatment plans.

Statistical analysis

A descriptive analysis was carried out to evaluate the data. Numerical variables were presented using mean and standard deviation (SD) if normally distributed. If not normally distributed, median and interquartile range (IQR) were used. Categorical variables were presented using count and percentage. One-sample t-test was used to compare the actual rate of ADA compliance to the expected rate (100%). The magnitude of compliance to the ADA guidelines was compared between the sub-groups using the Chi-squared test for categorical variables and independent samples t-test for normally distributed numerical variables. For numerical variables not normally distributed, values were compared between the sub-groups using the independent samples Mann-Whitney U test. All tests were conducted by considering a 0.05 level of significance. Statistical analysis was carried out using IBM SPSS Statistics version 25 (IBM Corp., Armonk, NY).

## Results

The study included 202 type 2 diabetic patients (Table 1), of which two-thirds were male (73.3%) and one-third were female (26.7%); the mean age of the patients was 60 years. The mean HbA1c level was 9.4 ± 1.9% and the mean duration of diabetes was 8.5 ± 5.0 years. All patients had moderately elevated albuminuria (43) and a mean eGFR of 29. 1 ± 9.1 mL/min/1.73m^2^. Patients' weight ranged from normal to obese with a mean BMI of 29.1 ± 9.1 kg/m^2^ and a mean LDL level of 2.7 ± 1.24 mmol/L. It is worth mentioning that although all the patients included in this study had either established ASCVD or were at high risk of developing it, 86 (42.6%) patients had underlying heart failure, of which 42% had reduced ejection fraction while the remaining had preserved ejection fraction.

**Table 1:** Demographic and clinical data of patients included in the study (n=202) BMI: body mass index; eGFR: estimated glomerular filtration rate; ACR: albumin-to-creatinine ratio; IQR: interquartile range; LDL: low-density lipoprotein; HDL: high-density lipoprotein; HbA1c: hemoglobin A1c; ASCVD: atherosclerotic cardiovascular disease; HFrEF: heart failure with reduced ejection fraction; HFpEF: heart failure with preserved ejection fraction

Study variables	Values
Age, years, mean ± SD	59.8 ± 9.8
Gender, n (%)	
Male	148 (73.3%)
Female	54 (26.7%)
Nationality, n (%)	
Saudi	89 (44.1%)
Non-Saudi	113 (55.9%)
BMI, kg/m^2^, mean ± SD	29. 1 ± 9.1
Systolic blood pressure, mmHg, mean ± SD	131.2 ± 15.2
Diastolic blood pressure, mmHg, mean ± SD	73.5 ± 11.9
Laboratory results	
eGFR, mL/min/1.73m^2^,mean ± SD	74.2 ± 27.4
ACR, mg/g, median (IQR)	43 (189)
LDL, mmol/L, mean ± SD	2.7 ± 1.24
HDL, mmol/L, mean ± SD	0.952 ± 0.27
Cholesterol, mmol/L, mean ± SD	3.97 ± 1.3
Triglycerides, mmol/L, mean ± SD	1.79 ± 1.16
Status of diabetes mellitus, mean ± SD	
Duration of diabetes, years	8.5 ± 5.0
HbA1c, %	9.14 ± 2.0
Mean HbA1c, %	9.4 ± 1.9
Cardiovascular disease/risk status, n (%)	
Established/high risk of ASCVD	202 (100.0%)
Heart failure, n (%)	
Present	86 (42.6%)
Absent	116 (57.4%)
Ejection fraction among patients with heart failure, n (%)	
HFrEF	36 (41.9%)
HFpEF	50 (58.1%)
Ejection fraction, %, mean ± SD	44.5 ± 13.7

**Figure 1:**
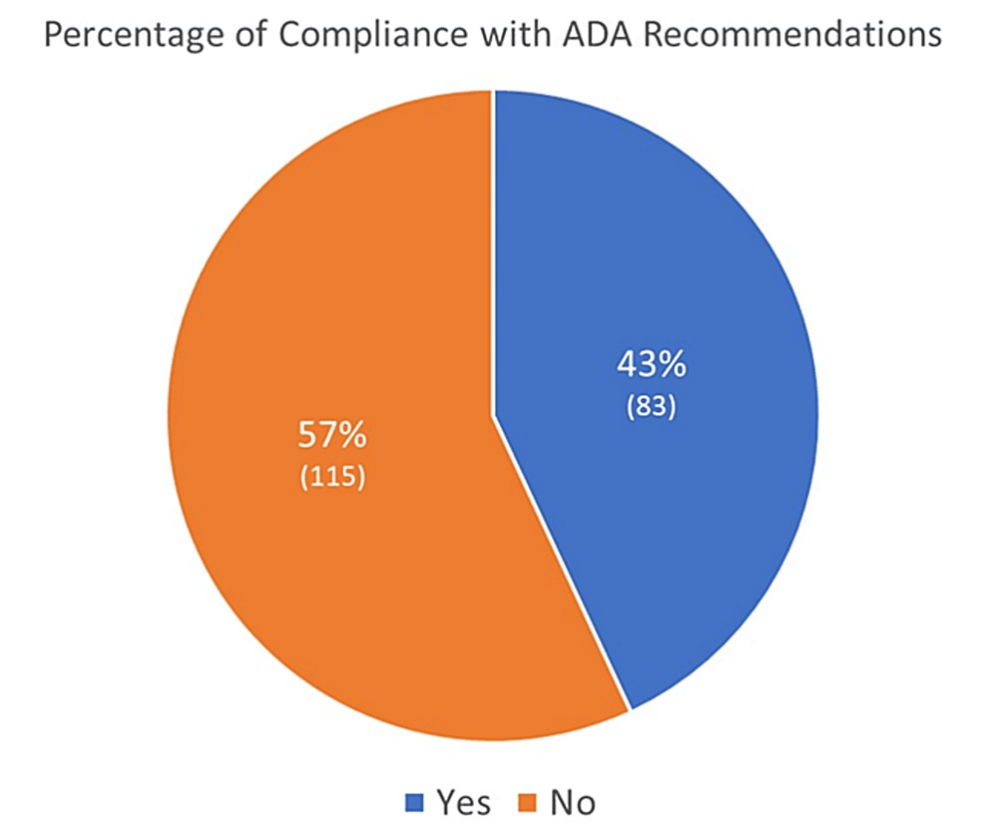
Compliance with ADA recommendations in terms of prescribing cardioprotective antihyperglycemic medications to eligible diabetic patients ADA: American Diabetes Association

Given that all patients in this study had established/high-risk ASCVD, all of them should have been placed on cardioprotective medications in compliance with ADA recommendations. However, a one-sample t-test revealed that the percentage of compliance with the recommendations (57%), as illustrated in Figure 1, was significantly lower than 100% (p<0.001).

Analyzing it further, physicians' adherence to the recommendations in prescribing medications was compared particularly between patients with heart failure and those without by using the Chi-square test at a 0.05 level of significance. As shown in Table 2, an insignificant difference in practice was noted, with only about half (48, 55.8%) of the total number of patients with heart failure (86) being placed on a cardioprotective antihyperglycemic agent (p=0.783).

**Table 2:** Physicians’ compliance with ADA recommendations in prescribing cardioprotective antihyperglycemic medications to patients with and without heart failure ADA: American Diabetes Association

Heart failure	Prescribed medications				
	Yes (115)		No (87)		P-value
	N	%	N	%	
Present	48	55.8	38	44.2	0.783
Absent	67	57.8	49	42.2	

Similarly, as shown in Table 3, the level of ejection fraction among heart failure patients did not appear to significantly affect physicians' decisions in treating them with the medications. Only 55.6% of HFrEF patients and 68% of HFpEF patients were prescribed medication(s) (p=0.239).

**Table 3:** Physicians’ compliance with ADA recommendations in prescribing cardioprotective antihyperglycemic medications to those with reduced/preserved ejection fraction among patients with heart failure HFrEF: heart failure with reduced ejection fraction; HFpEF: heart failure with preserved ejection fraction; ADA: American Diabetes Association

Ejection fraction among 86 patients with heart failure	Prescribed medications				
	Yes (54)		No (32)		P-value
	N	%	N	%	
HFrEF	20	55.6	16	44.4	0.239
HFpEF	34	68.0	16	32.0	

## Discussion

In this retrospective records review conducted at King Abdulaziz University Hospital in Jeddah, we evaluated physicians' adherence to the ADA treatment guidelines for adults with type 2 diabetes and underlying cardiovascular disease(s). All patients included in this study were eligible for the prescription of cardioprotective medications (SGLT2 inhibitors and GLP1-RAs) as per the guidelines. However, almost half of the patients had not been prescribed either medication, showing a 43% non-adherence rate with regard to the ADA guidelines (p<0.001). Furthermore, the presence of heart failure with reduced or preserved ejection fraction did not seem to significantly influence physicians' decision to prescribe these medications.

These findings are relatively better compared to the results of the CAPTURE study conducted in 2019 across several countries including Saudi Arabia, where only one in four patients (23.6%) with type 2 diabetes and established CVD were found to be on a cardioprotective antihyperglycemic medication. However, they still fall significantly short in their adherence to guidelines that were framed to help reduce the burden of CVD in people with type 2 diabetes. These results could be attributed to limitations related to individual purchasing decisions, policies, and prescription privileges held by each government hospital in the Kingdom of Saudi Arabia affecting the availability of these medications [6]. Interestingly, at King Abdulaziz University Hospital, a government health institute, prescribing SGLT2 inhibitors or GLP1-RAs is not restricted to a certain specialization, but the disruption in the availability of these medications may be a factor influencing the lack of adherence.

As is well known, glycemic control is a strong predictor of long-term diabetes complications. In spite of that, less stringent blood glucose control is recommended in patients with ischemic heart disease, since strict control among them may lead to an increase in mortality, as was observed in the ACCORD trial [23]. Being mindful of this fact and the state of the patients, poorly controlled glycemic levels were found in this study with a mean HbA1c level of 9.14 ± 2%. This finding is quite similar to the results of Radi et al. published in 2016, where the achievement of treatment goals in type 2 diabetes among 201 patients in the same university hospital was evaluated and showed a mean HbA1c of 8.67 ± 2.35%, with only 24% of the study population achieving treatment target [24]. It is possible that a less stringent blood glucose control may have been advised for these patients, but a mean HbA1c of 9.4% is still questionable.

As recommended by the ADA, target blood pressure levels in individuals with diabetes and existing ASCVD or a high 10-year ASCVD risk should be below 130/80 mmHg if attained safely [22]. In light of this, it is gratifying to see that the mean systolic and diastolic blood pressure levels in our study (131.2 ± 15.2 and 73.5 ± 11.9 mmHg respectively) were overall close to the target levels, whereas only 29.03% achieved target BP levels in the study by Radi et al. (mean systolic BP: 143.3 ± 27 mmHg, mean diastolic BP: 75.5 ± 12.9 mmHg) [24].

Despite the clear recommendation for strict LDL control in patients with established or at high risk of ASCVD, it is notable that the mean LDL levels found in this study group (2.7 ± 1.24 mmol/L) are not in line with these recommendations [22]. The same was the case in the study by Radi et al. in 2016 (mean LDL: 2.67 ± 0.98 mmol/L), indicating that there has been no improvement in the situation after six years [24].

It is important to bear in mind that this study was carried out immediately after the coronavirus disease 2019 (COVID-19) pandemic, due to which many follow-up appointments had been canceled or rescheduled, leading to long waiting periods and a gap in the continuity of care. Another factor that could be attributed to our disappointing findings, as echoed by Radi et al., could be the poor socioeconomic status of the patients treated at this university hospital, making them unable to effectively follow prescriptions and purchase more effective and newer medications [24]. All the aforementioned factors undoubtedly have an impact on diabetes control and a physician’s treatment plan, posing a potential obstacle in adhering to treatment guidelines and prescribing ideal medications.

## Conclusions

Overall, the results of this study show a lack of adherence to treatment guidelines, which needs to be taken seriously given the complications such patients may suffer and the consequent burden on the health system. Physicians need to be kept abreast of the most recent guidelines on the use of cardioprotective antihyperglycemic medications and their benefits via workshops and lectures. In addition, it is advisable to make such medications available with a support plan for patients of low socioeconomic status. Furthermore, we recommend further research to identify the reasons behind the lack of adherence to guidelines by physicians and the obstacles they face in implementing them so that they can be properly addressed to achieve the desired treatment goals effectively.
